# Genetic enhancement of Ras-ERK pathway does not aggravate L-DOPA-induced dyskinesia in mice but prevents the decrease induced by lovastatin

**DOI:** 10.1038/s41598-018-33713-3

**Published:** 2018-10-18

**Authors:** Irene Ruiz-DeDiego, Stefania Fasano, Oscar Solís, José-Rubén Garcia-Montes, José Brea, María I. Loza, Riccardo Brambilla, Rosario Moratalla

**Affiliations:** 1 Instituto Cajal, Consejo Superior de Investigaciones Científicas (CSIC), Madrid, Spain; 2 CIBERNED, Instituto de Salud Carlos III, Madrid, Spain; 3 Neuroscience and Mental Health Research Institute (NMHRI), Neuroscience Division, School of Biosciences, Cardiff University, Cardiff, United Kingdom; 4 Instituto de Farmacia Industrial, Fac de Farmacia, Universidad de Santiago de Compostela, Santiago de Compostela, Spain; 5 Centro de Investigación CIMUS, Universidad de Santiago de Compostela, Santiago de Compostela, Spain

## Abstract

Increasing evidence supports a close relationship between Ras-ERK1/2 activation in the striatum and L-DOPA-induced dyskinesia (LID). ERK1/2 activation by L-DOPA takes place through the crosstalk between D1R/AC/PKA/DARPP-32 pathway and NMDA/Ras pathway. Compelling genetic and pharmacological evidence indicates that Ras-ERK1/2 inhibition prevents LID onset and may even revert already established dyskinetic symptoms. However, it is currently unclear whether exacerbation of Ras-ERK1/2 activity in the striatum may further aggravate dyskinesia in experimental animal models. Here we took advantage of two genetic models in which Ras-ERK1/2 signaling is hyperactivated, the *Nf1*^+/−^ mice, in which the Ras inhibitor neurofibromin is reduced, and the Ras-GRF1 overexpressing (Ras-GRF1 OE) transgenic mice in which a specific neuronal activator of Ras is enhanced. *Nf1*^+/−^ and Ras-GRF1 OE mice were unilaterally lesioned with 6-OHDA and treated with an escalating L-DOPA dosing regimen. In addition, a subset of *Nf1*^+/−^ hemi-parkinsonian animals was also co-treated with the Ras inhibitor lovastatin. Our results revealed that *Nf1*^+/−^ and Ras-GRF1 OE mice displayed similar dyskinetic symptoms to their wild-type counterparts. This observation was confirmed by the lack of differences between mutant and wild-type mice in striatal molecular changes associated to LID (i.e., FosB, and pERK1/2 expression). Interestingly, attenuation of Ras activity with lovastatin does not weaken dyskinetic symptoms in *Nf1*^+/−^ mice. Altogether, these data suggest that ERK1/2-signaling activation in dyskinetic animals is maximal and does not require further genetic enhancement in the upstream Ras pathway. However, our data also demonstrate that such a genetic enhancement may reduce the efficacy of anti-dyskinetic drugs like lovastatin.

## Introduction

Parkinson’s disease (PD) is the second most common neurodegenerative disorder after Alzheimer’s disease. This progressive, chronic, and age-related pathology is characterized by a dramatic depletion of dopamine in the striatum following loss of dopaminergic neurons in the substantia nigra pars compacta. Despite extensive investigation aimed at finding new therapeutic approaches, the dopamine precursor molecule L-DOPA remains the most effective and widely used non-invasive therapy for PD. However, in the vast majority of patients, chronic treatment and disease progression cause the appearance of abnormal involuntary movements known as dyskinesias. L-DOPA-induced dyskinesia (LID) interferes significantly with normal motor activity and persists unless L-DOPA dosages are reduced below therapeutic levels. Thus, controlling dyskinetic symptoms is one of the major challenges in PD therapy^1,2^.

Extensive work indicates that extracellular signal-regulated kinase 1/2 (ERK1/2) activation in the striatum plays a critical role in the emergence of LID^1–12^. This activation takes place by two interplaying cascades: the D1R/cAMP/PKA/DARPP-32 pathway and the NMDA/Ras pathway. In the dopamine-depleted striatum, L-DOPA leads to D1R activation and stimulation of adenylyl cyclase through Gαolf protein^13^, increasing cAMP accumulation and activating PKA, modulating synaptic plasticity^14,15^. In turn, PKA phosphorylates DARPP-32 at Thr34^9,16–18^, which in its phosphorylated state acts as a potent inhibitor of protein phosphatase-1 (PP1). The inhibition of PP-1 thereby controls many downstream effectors, including activation of mitogen-activated protein kinase (MEK, which phosphorylates ERK1/2) and inhibition of STEP (which dephosphorylates ERK1/2)^19^. Importantly, genetic deletion or pharmacological blockade of D1R prevents the supersensitive ERK1/2 response and LID^4,20^.

In addition to D1R-pathway, the ERK1/2 cascade can also be activated by the NMDA receptor via Ras^21,22^. In this pathway, lovastatin, a drug widely used to treat hyperlipidemia and able to reduce Ras activity by inhibiting its isoprenylation, significantly attenuates the phosphorylation of ERK1/2 and the development of LID^23,24^. In addition, genetic inactivation of the Ras-guanine nucleotide-releasing factor 1 (Ras-GRF1), a neuronal specific activator of Ras, attenuates dyskinesia in mice and non-human primates^3,5^.

The neurofibromatosis type 1 (*Nf1)* gene codes for neurofibromin, a cytoplasmic protein that functions as a GTPase-activating protein for Ras^25,26^. Neurofibromin inhibits Ras activity by catalyzing the conversion of Ras from its active GTP-bound (Ras-GTP) to its inactive GDP-bound form^27,28^. Reduced neurofibromin expression by *Nf1* gene mutations is associated with increased Ras-ERK1/2 activation^29,30^ and, in the brain, with learning and memory deficits^27,31–33^. Interestingly, pharmacological inhibition of Ras activity with lovastatin ameliorates the observed behavioral abnormalities in mice and humans^34–36^. In the present work, we investigated the role of ERK1/2 over-activation in LID with two different approaches: a “direct” approach using Ras-GRF1 overexpressing (Ras-GRF1 OE) transgenic mice, and an “indirect” intervention, favoring the maintenance of Ras in its active form by decreasing the neurofibromin levels using *Nf1*^+/−^ transgenic animals.

## Results

### Basal locomotor activity and motor coordination are normal in *Nf1*^+/−^ mutant mice

**Figure 1:**
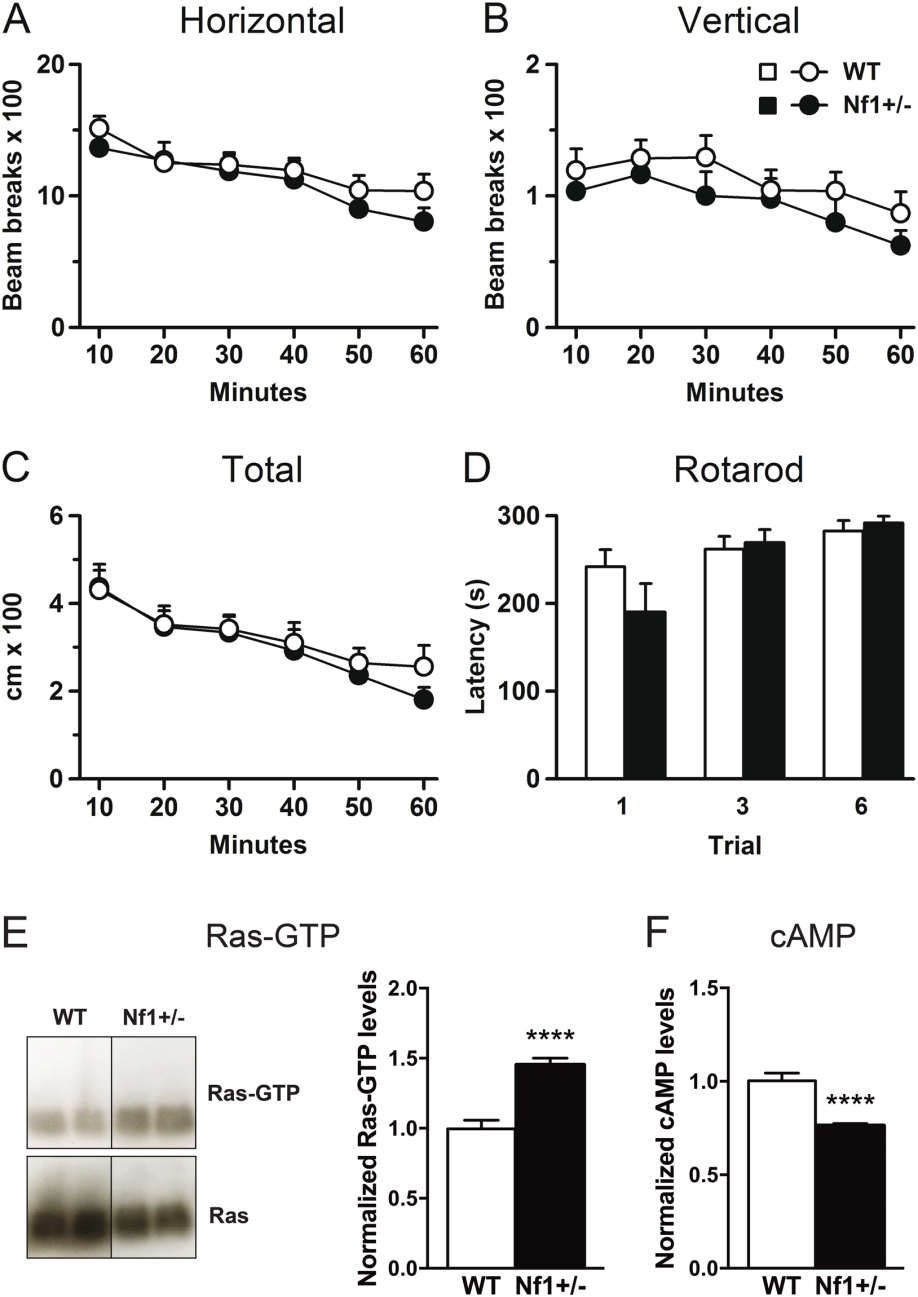
Heterozygous *Nf1* gene mutation does not affect basal locomotor activity but increased striatal Ras-GTP levels. (**A**) Horizontal and (**B**) vertical activity and (**C**) total distance traveled measured for 60 min in a multicage activity meter system. (**D**) Motor coordination measured in the rotarod at constant acceleration and expressed as latency to fall from the rotating rod. Striatal Ras-GTP levels determined by WB analysis are increased in *Nf1*^+/−^ naïve mice (**E**) while cAMP levels are decreased compared to their WT counterparts (**F**). Uncropped blots are provided in Suplementary Fig. S1. ^****^p < 0.0001 vs WT. n = 7–12.

In order to rule out any baseline motor impairment in the mutant mice, we carried out spontaneous locomotor activity and rotarod tests prior to the 6-OHDA lesion. Since previous experiments from Fasano and colleagues^37^ demonstrated that the increase of Ras-GRF1 expression in Ras-GRF1 OE mice does not affect locomotor activity and motor coordination in these animals, we focused on testing these behavioral performances in *Nf1*^+/−^ mice. *Nf1*^+/−^ and wild-type (WT) mice showed similar horizontal and vertical activity, as indicated by the number of beam breaks measured in a multi-cage activity meter system (Fig. 1A,B). Ambulatory activity measured as total distance traveled was also similar in both genotypes (Fig. 1C). Likewise, there were no statistically significant differences between genotypes in motor coordination (Fig. 1D), measured as the latency to fall from the rotarod. These results indicated that alterations in *Nf1* gene expression do not modify spontaneous locomotor activity or motor coordination.

### *Nf1*^+/−^ mutant mice show high striatal Ras-GTP levels but reduced cAMP levels

Next, to confirm that *Nf1*^+/−^ mice showed a higher activation of the Ras-ERK1/2 signaling pathway, we studied striatal Ras-GTP levels in naïve animals. As expected, we found that *Nf1*^+/−^ mice displayed higher levels of Ras-GTP compared to WT mice (Fig. 1E and supplementary Fig. S1). We also examined whether the mutation in neurofibromin could modulate the activity of Gαolf protein by testing cAMP levels in the striatum. Interestingly, we found a decrease in cAMP levels in *Nf1*^+/−^ mutant mice relative to WT counterparts (Fig. 1F).

### Heterozygous deletion of *Nf1* has no effect on L-DOPA-induced dyskinesia or LID-associated changes in gene expression

To explore the role of *Nf1* in dyskinesia, we studied the development of LID in hemiparkinsonian/6-OHDA-lesioned mice by scoring axial dystonia and forelimb and orofacial dyskinesias in *Nf1*^+/−^ and their WT littermates. Since high L-DOPA doses can mask subtle differences between genotypes, we used an escalating dosing regimen in which L-DOPA was given i.p. daily for 9 days, with the daily dose increasing over the course of treatment (5, 10, and 20 mg/kg i.p., 3 consecutive days at each dose).

**Figure 2:**
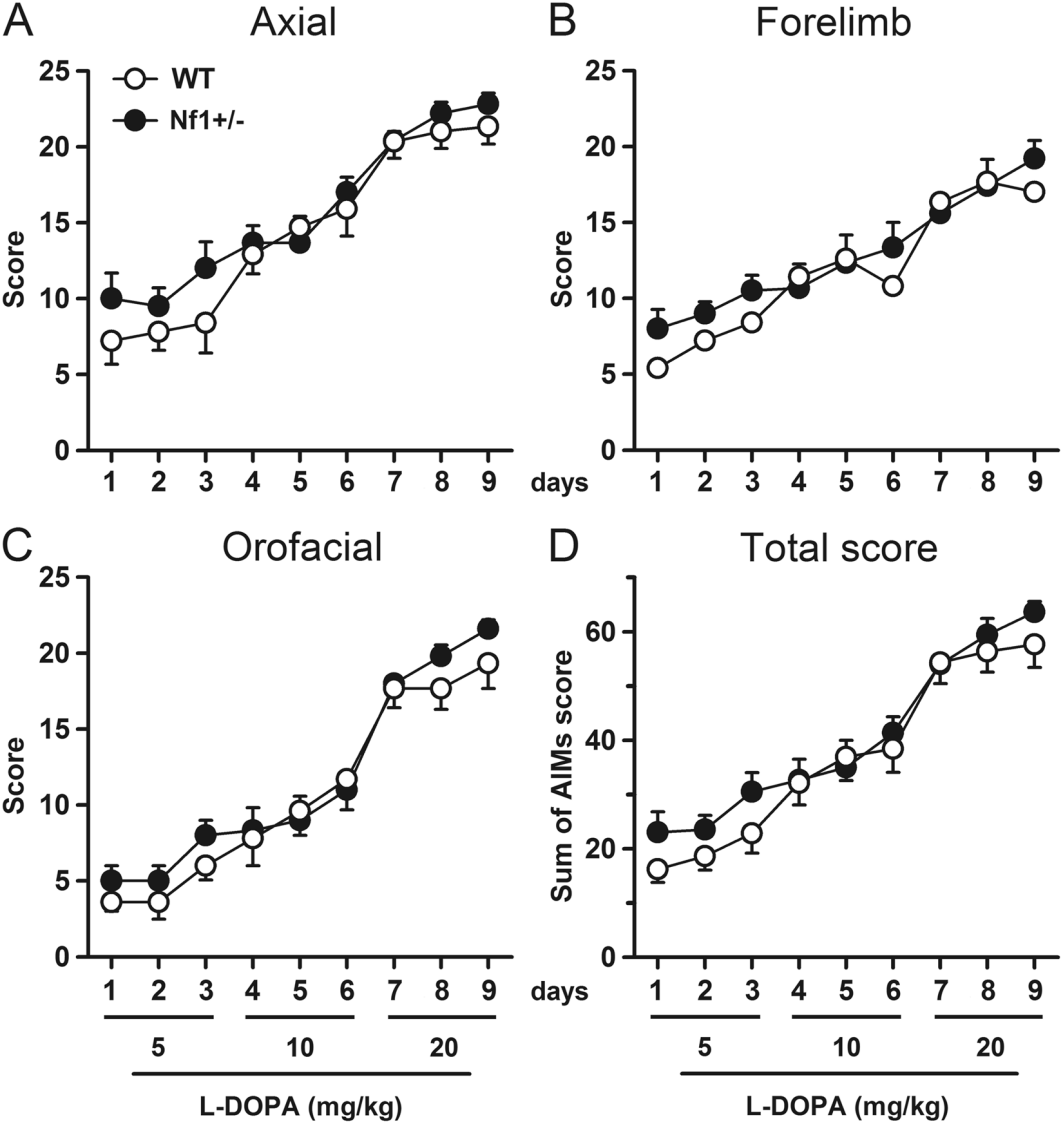
Heterozygous *Nf1* gene mutation does not modify dyskinetic behaviour induced by L-DOPA. Temporal profile of **(A)** axial dystonia, **(B)** forelimb dyskinesia, **(C)** orofacial dyskinesia and **(D)** cumulated total score induced by an escalating L-DOPA dosing regimen administered in 9 consecutive days. No differences were found between *Nf1*^+/−^ mice and their littermate controls. n = 5–9.

Daily scoring of AIMs (abnormal involuntary movements, i.e., axial dystonia, forelimb and orofacial dyskinesia) revealed a gradual development of dyskinesia in both genotypes, with AIMs scores increasing as the L-DOPA dose ascended. Interestingly, heterozygous *Nf1* mutation had no effect on dyskinetic scores at any of the three L-DOPA doses: there were no significant differences in total dyskinetic scores between *Nf1*^+/−^ and WT mice across the 9-day L-DOPA treatment (Fig. 2D). Both transgenic and WT mice showed great lateral deviation, twisted posture, contralateral forelimb dyskinesia, and jaw movements with tongue protrusion, with no statistically significant differences between genotypes for any of the three AIMs subtypes (Fig. 2A,C).

**Figure 3:**
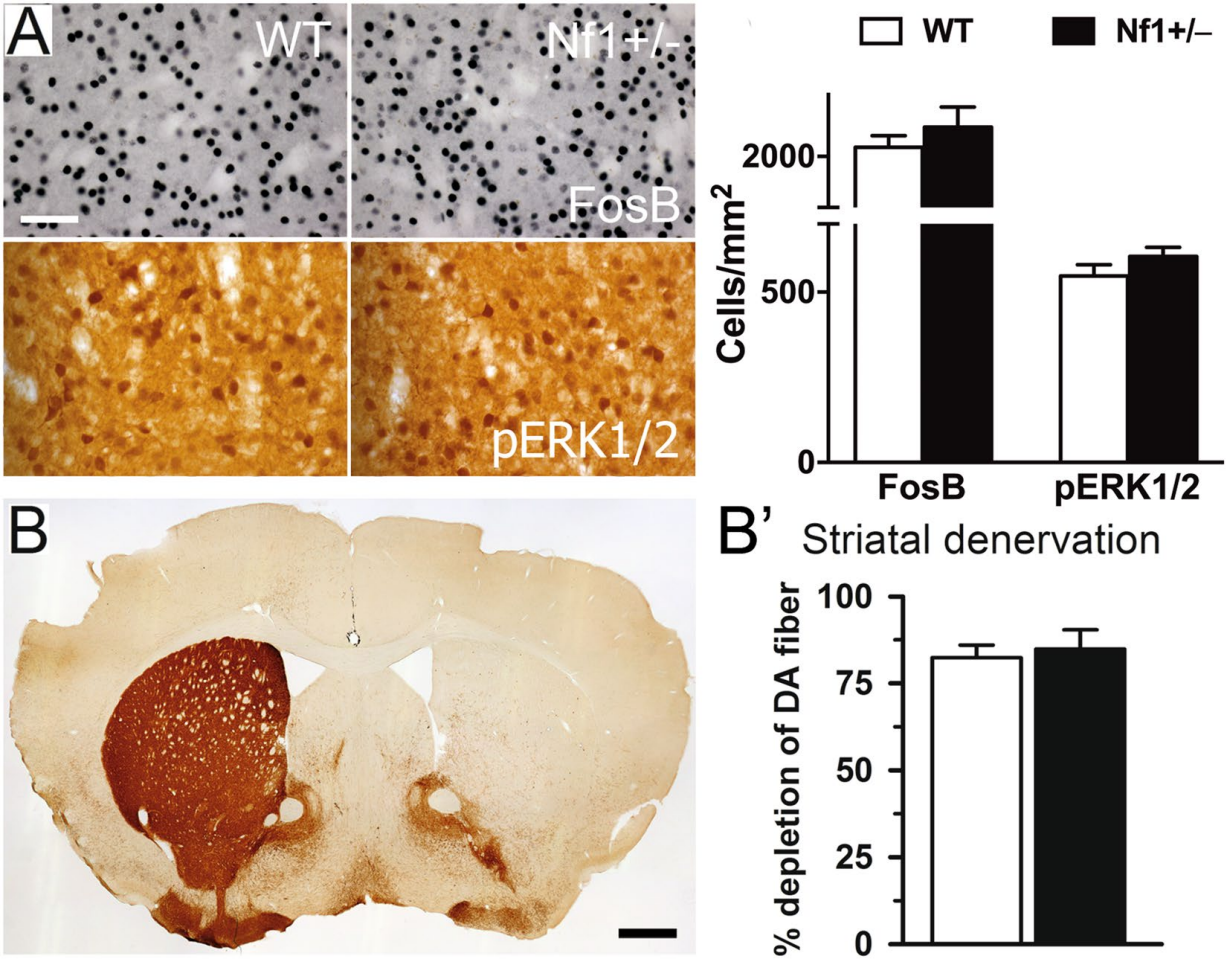
Heterozygous *Nf1* gene mutation does not alter molecular changes induced by L-DOPA. (**A**) High-power photomicrographs of denervated striatum from WT and heterozygous *Nf1* mice immunostained for FosB and pERK1/2. Histogram represents the quantification of FosB and pERK1/2 immunoreactive positive nuclei. (**B**) Bar graphs show the percentage of depletion of dopaminergic fibers measured in the striatum. No differences were found between *Nf1*^+/−^ and WT mice. n = 5–9. Scale bars = 50 µm, 500 µm.

Correlating with these behavioral results, analysis of the dorsolateral striatum showed that L-DOPA-induced expression of FosB and pERK1/2 were similar in both genotypes (Fig. 3A), as well as pAcH3 expression (data not shown). The percentage of denervated striatum also demonstrated no different denervation between groups (Fig. 3B,B′).

### Overexpression of Ras-GRF1 does not affect L-DOPA-induced dyskinesia

**Figure 4:**
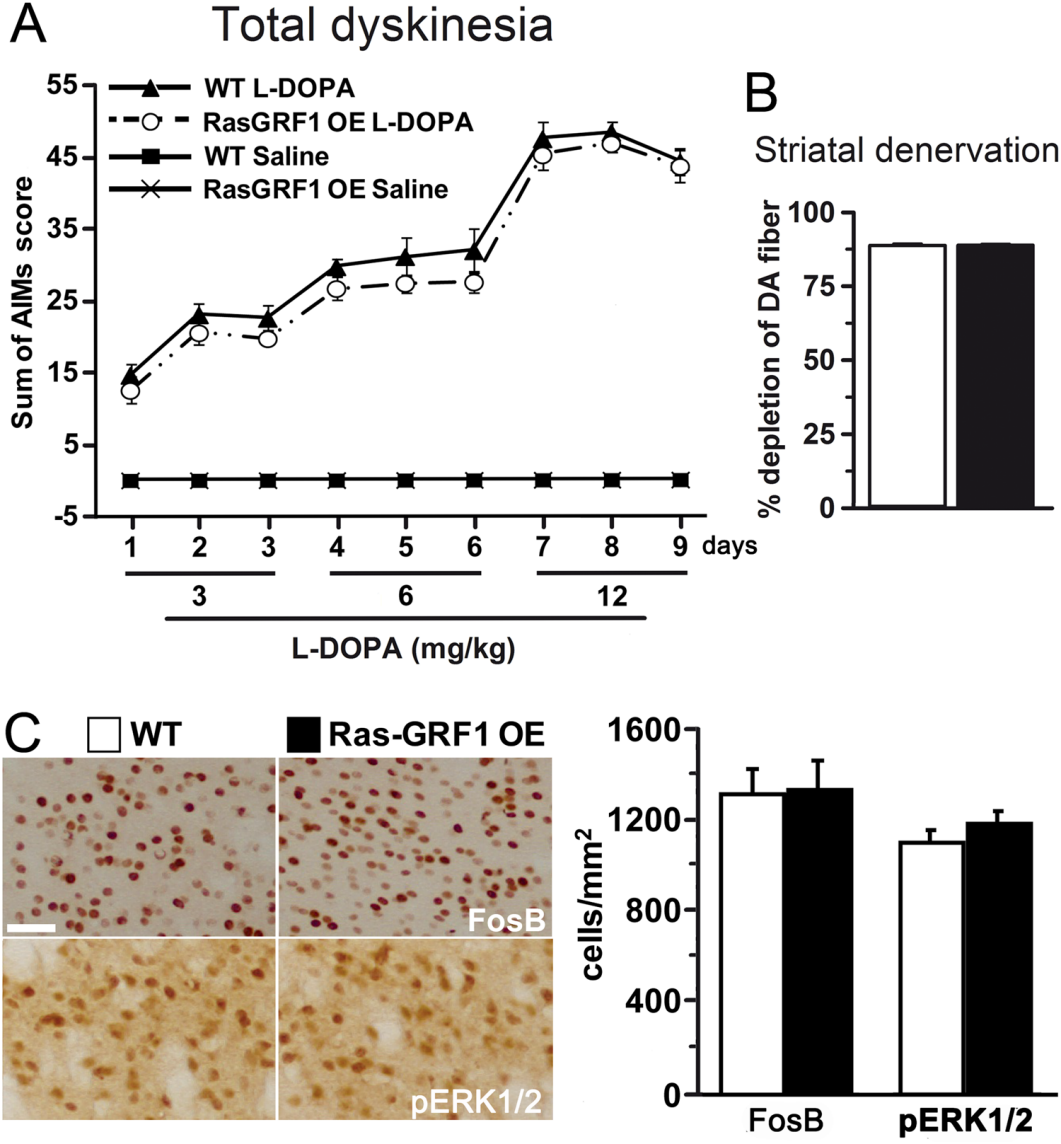
Overexpression of Ras-GRF1 does not alter dyskinetic symptoms or the associated molecular markers induced by L-DOPA. (**A**) Temporal profile of axial, limb and orofacial AIMs induced by an escalating L-DOPA dosing regimen administered in 9 consecutive days. **(B)** Histograms show the percentage of striatal TH immunoreactive fibers depletion as a measure of dopaminergic denervation. (**C**) High-power photomicrographs of striatum from WT and Ras-GRF1 OE mice immunostained for FosB and pERK1/2. Bar graphs represent the quantification of FosB and pERK1/2 immunoreactive positive nuclei. No differences were found between transgenic Ras-GRF1 OE mice and their littermate controls. n = 10–12. Scale bar = 50 µm.

We next explored the effects of direct intervention in Ras signaling by using Ras-GRF1 overexpressing (Ras-GRF1 OE) mice^37,38^. Ras-GRF1 protein levels in these animals are threefold higher than in WT. Importantly, we previously shown that Ras-GRF1 OE mice are more sensitive to cocaine as measured by enhanced ERK1/2 activation in the striatum and increased behavioral responses^37^. Similarly to *Nf1*^+/−^ mice, Ras-GRF1 OE animals showed dyskinetic symptoms upon an escalating dose protocol comparable to WT controls (Fig. 4A). Accordingly, both the percentage of denervated striatum and the striatal expression of pERK1/2 and FosB were indistinguishable between Ras-GRF1 OE and WT mice (Fig. 4B,C).

### Lovastatin attenuates L-DOPA-induced dyskinesia severity in WT but not in *Nf1*^+/−^ mice

**Figure 5:**
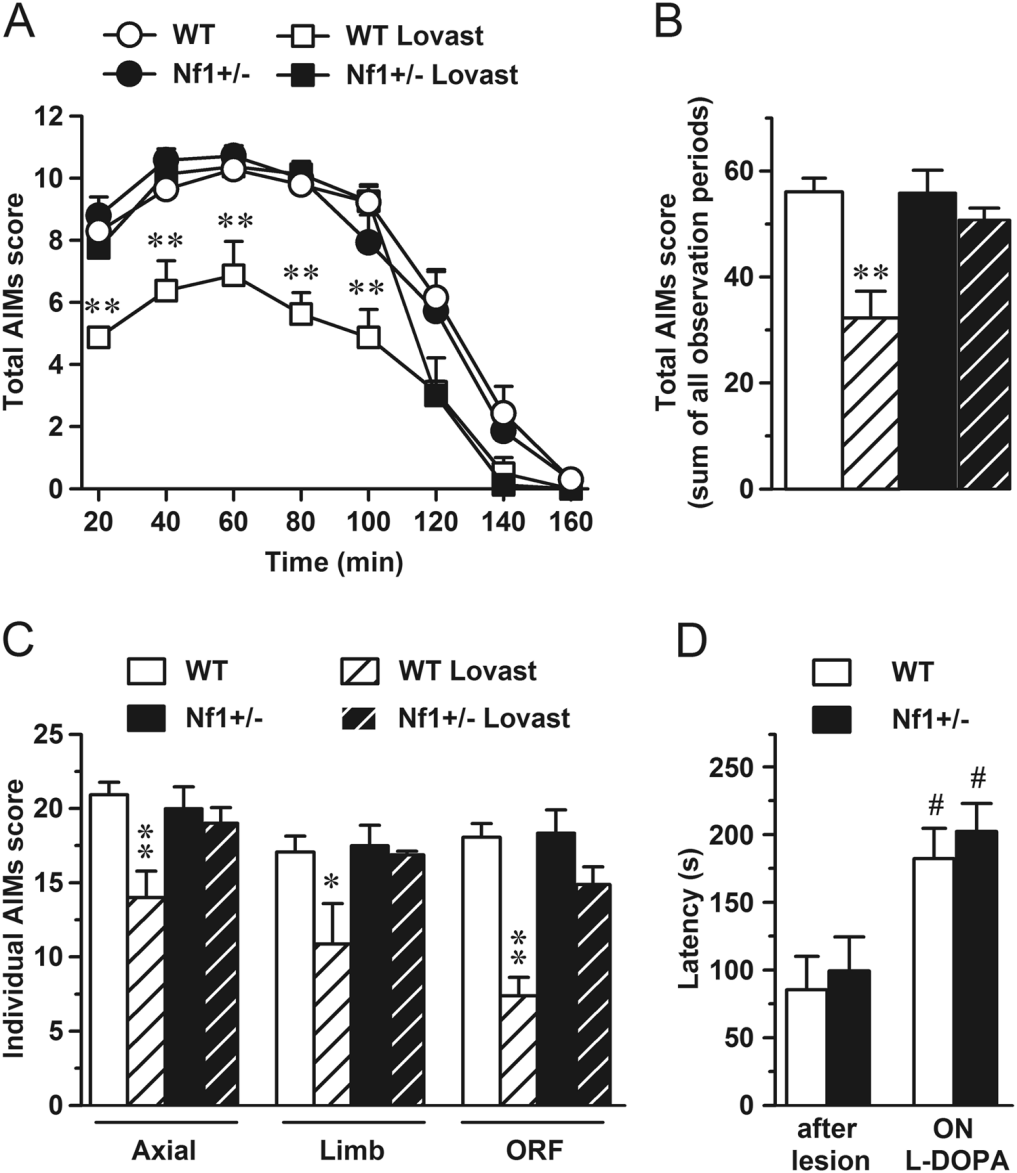
Chronic lovastatin treatment reduces AIMs scores in WT but not in heterozygous *Nf1* mice. (**A**) Time profile of AIMs evaluated for 1 min every 20 min during 160 min on last day of L-DOPA treatment. (**B**) Sum of total AIMs score during all observation periods. (**C**) Sum of all AIM subscores (axial, limb and orofacial) evaluated during all observation periods. (**D**) Motor coordination assessed of hemiparkinsonian mice before L-DOPA treatment (after lesion) and on day 14, 24 h after L-DOPA administration (ON L-DOPA) to verify that genetic manipulation of *Nf1* does not modify the therapeutic effect of L-DOPA. *p = 0.01, ^**^p < 0.001 vs WT. ^#^*p* < 0.001 vs *after lesion* situation. n = 4–8.

To assess the effect of pharmacological blockade of Ras on LID we used lovastatin, a Ras activity inhibitor. Mice were pretreated with lovastatin (20 mg/kg i.p.) or 0.9% saline for 3 days, as previously described^23^. Afterwards, for 10 consecutive days, mice received 20 mg/kg of L-DOPA 2.5 h after lovastatin or saline administration. The time course of AIMs expression following 20 mg/kg L-DOPA treatment was similar in *Nf1*^+/−^ and WT mice (Fig. 5A). Dyskinesia was evident at 20 minutes, peaked between 40 and 60 minutes, declined significantly by 100–120 minutes, and disappeared after 140–160 minutes for both genotypes. WT mice treated with both drugs exhibited significantly less dyskinesia than those treated with L-DOPA only. In contrast, lovastatin had no effect on the intensity of LID in *Nf1*^+/−^ transgenic mice. This difference was reflected in cumulative AIMs scores over the entire 160-min observation period (Fig. 5B). Those scores were reduced by lovastatin in WT mice but not in *Nf1*^+/−^. Analysis of the individual AIMs over the entire 160-min observation revealed that the effect of lovastatin in WT mice was evident in all the dyskinetic sub-symptoms (Fig. 5C). Intriguingly, lovastatin did not modify frequency or intensity of any individual dyskinetic symptoms in the *Nf1*^+/−^ mutant mice.

To exclude any possible interaction of *Nf1* mutation on the antiparkinsonian properties of L-DOPA, we performed the rotarod test before and after the L-DOPA treatment. In the absence of L-DOPA, parkinsonian animals of both WT and *Nf1*^+/−^ genotypes showed similar latencies to fall from the rotating rod (Fig. 5D), while under chronic L-DOPA treatment both groups of mice showed an improved motor performance, indicating a similar therapeutic response to L-DOPA.

## Discussion

The major finding of this study is that the onset and expression of LID in *Nf1*^+/−^ and in Ras-GRF1 OE mice are indistinguishable from WT. This indicates that a genetic enhancement of the Ras-ERK1/2 pathway does not result in either an exacerbated dyskinetic behavior or in the increase of the associated molecular markers (i.e. FosB, pERK1/2 and pAcH3 expression). Moreover, we demonstrate that pharmacological inhibition of Ras function with lovastatin decreases dyskinetic signs in WT mice, although is unable to reduce LID in the *Nf1*^+/−^ mice.

Since previous works indicate that there is a correlation between Ras-ERK1/2 activity levels and intensity of dyskinetic symptoms^1,2,6,8,39^, we expected that *Nf1*^+/−^ and Ras-GRF1 OE mice would exhibit more dyskinesia than WT animals. However, this increase in dyskinetic symptoms is not evident in our transgenic mice, nor is an increase in the molecular changes induced by L-DOPA.

**Figure 6:**
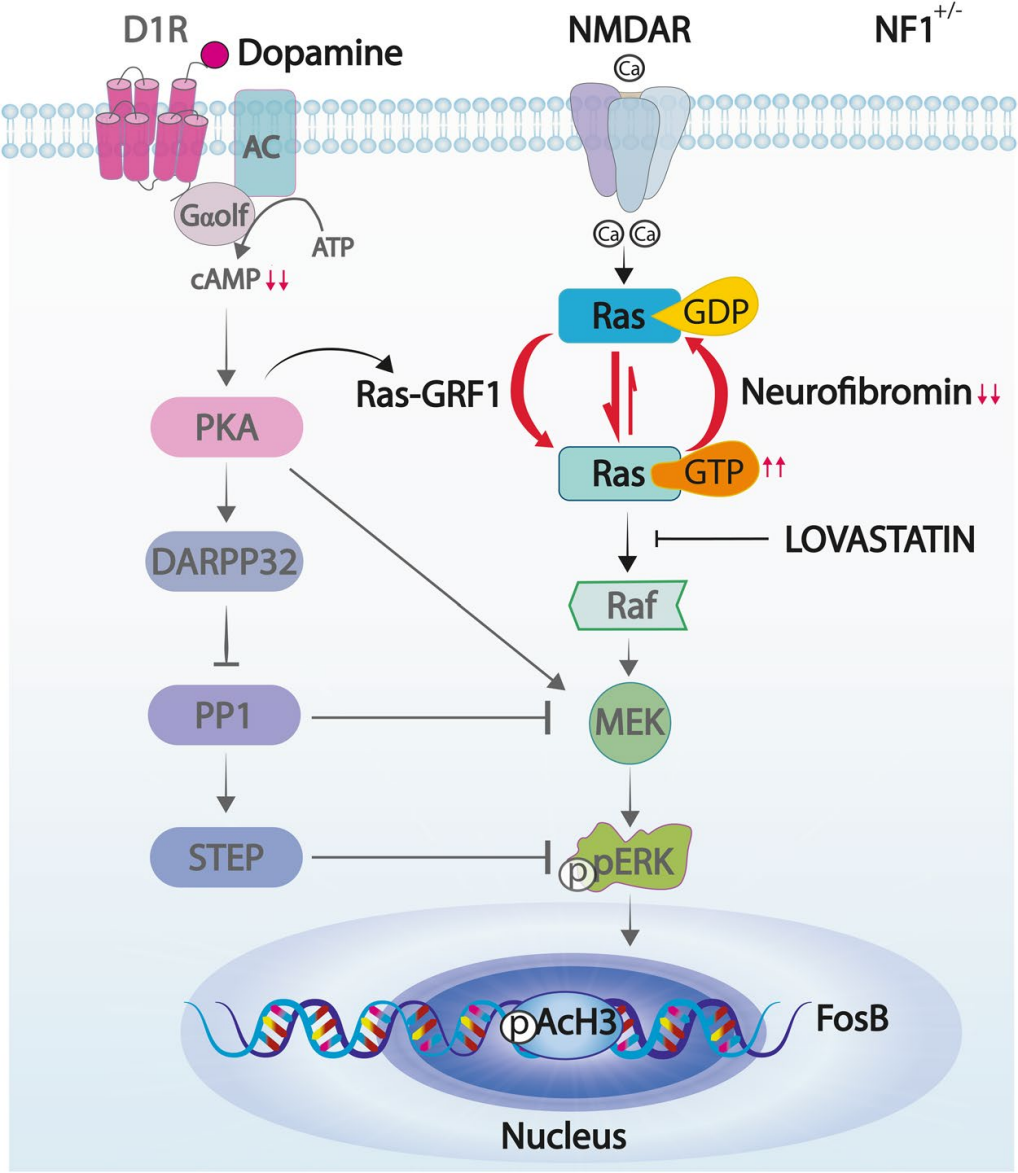
Schematic diagram illustrating D1R and NMDAR signaling cascades in heterozygous *Nf1* mice. In the striatum, dopamine activates D1R leading to cAMP formation and PKA activation that in turn leads to ERK1/2 phosphorylation. In addition, stimulation of NMDAR promotes Ras activation through Ras-GRF1 which catalyzes the conversion of inactive Ras-GDP to active Ras-GTP. This process is counteracted by neurofibromin, which diminishes Ras activity. However, in heterozygosis (*Nf1*^+/−^) the balance between Ras-GTP and Ras-GDP is displaced towards the active form, favoring ERK1/2 phosphorylation while cAMP levels are reduced.

As expected, *Nf1*^+/−^ mice showed higher striatal Ras-GTP levels compared to their WT littermates, due to the heterozygous deletion of *Nf1* gene. In heterozygosis, neurofibromin is less efficient at converting Ras-GTP to its inactive form Ras-GDP, leading to higher levels of Ras-GTP as depicted in Fig. 6. On the other hand, this increase in the Ras-ERK1/2 signaling pathway seems to be counterbalanced by a decrease in the Gαolf-cAMP pathway, indicated by the lower cAMP striatal levels we found in *Nf1*^+/−^ mice. These results are in line with those found in both *Nf1* patient-derived induced pluripotent stem cell-neural progenitor cells and mouse *Nf1*^+/−^ hippocampal neurons^40^. Thus, we speculate that dyskinetic *Nf1*^+/−^ mice have the Ras-ERK1/2 pathway increased and the Gαolf-cAMP pathway decreased in the striatum, generating a net effect similar to that found in WT animals and is responsible for the same dyskinetic behavior displayed. Moreover, a similar scenario could be found in the Ras-GRF1 OE mice since these animals have also increased Ras activity similar to that of the *Nf1*^+/−^ mice. Future experiments to examine striatal cAMP activity in these mice may corroborate this idea.

Despite these negative results, we found that decreasing Ras activity with lovastatin decreases LID in WT mice but not in *Nf1*^+/−^ mice. Chronic co-treatment with lovastatin and L-DOPA effectively decreases dyskinetic symptoms in WT mice, consistent with previous work in hemiparkinsonian rats^23^. In our study, we demonstrate that the lovastatin-induced Ras-signaling pathway inhibition significantly reduced all AIMs in WT mice. These results are in line with previous findings using Ras-GRF1 knockout mice^5^, which also showed a decrease in LID. Taken together, this indicates that a direct inhibition in the Ras-ERK1/2 pathway is enough to reduce LID symptoms, but not to abolish them, as occurs when the D1R pathway is altered by a genetic ablation of D1R^4^. NMDA receptor antagonists do not modify ERK1/2 phosphorylation induced by D1R agonists in the denervated striatum^41^. Thus, these data suggest that D1R plays a more important role than the NMDA receptor in the control of ERK cascade following chronic L-DOPA treatment. However, it is also important to consider that metabotropic glutamate receptor type 5 (mGluR5) modulates D1R signaling pathway via ERK1/2 activation in the denervated striatum^42–44^. For instance, local infusion of a mGluR5 antagonist into the denervated striatum in hemiparkinsonian rats reduced D1R-agonist induced ERK1/2 signaling^42^.

In contrast with that found in WT animals, chronic administration of lovastatin did not diminish LID in transgenic *Nf1*^+/−^ mice, which have upregulated Ras function. Thus, lovastatin Ras-inhibition is not sufficient to reduce dyskinetic symptoms in these mice. These results are in line with previous findings which showed that Ras inhibitors can rescue only some phenotypes in *Nf1*-deficient cell lines^45^ and that lovastatin cannot correct the behavioral or the PET imaging deficits in mice^46^. More recently, Payne and colleagues^47^ performed the last and largest statin study in children with NF1 and concluded that lovastatin is not an effective treatment for cognitive or behavioral deficits in this population. At the signaling level, previous work *in vitro* with hippocampal neurons from *Nf1*^+/−^ mice showed that continuous lovastatin exposure normalizes both the attenuated cAMP levels and increased Ras activity in these mutant cells^40^. Thus, lovastatin decreases LID in WT animals by decreasing Ras activity, while in *Nf1*^+/−^ mice, although it decreases Ras activity, does not change LID, possibly because it may increase cAMP with a null net effect.

Finally, previous experiments demonstrated that the increased Ras-GRF1 expression in Ras-GRF1 OE mice does not affect locomotor activity and motor coordination^37^. Here we tested these behaviors in *Nf1*^+/−^ mice and found that these animals are indistinguishable from their WT littermates in motor activity and motor coordination, in agreement with previous observations in the open field test^33^ and the water maze focusing on swimming speed and other motor skills^33^ (for review see^48^). Since spontaneous locomotor activity and motor coordination are normal in the *Nf1*^+/−^ mice, results of dyskinesia experiments are not due to motor impairment, but rather to the modification of *Nf1*-related molecular pathways. Importantly, genetic modification in *Nf1*^+/−^ mice does not alter the kinetic profile of L-DOPA, as all animals show similar duration of the L-DOPA response and display similar performance skills in the rotarod test, demonstrating that the antiparkinsonian efficacy of L-DOPA is preserved in all the animals.

In conclusion, it appears from the present study that a plateau in ERK1/2-signaling activation may already be achieved in response to denervation and L-DOPA treatment, therefore a genetic enhancement of the Ras pathway does not exacerbate dyskinetic behavior. However, the lack of anti-dyskinetic effect in response to lovastatin observed in mice with abnormally high Ras activity may be of clinical significance since patients affected by such condition – RASopathies - do exist and may be tested in this respect in the near future.

## Methods and Materials

### Animals

Since the *Nf1* null mouse is lethal^35,49^, we used heterozygous mice for the *Nf1* gene (*Nf1*^+/−^)^27^ obtained by heterozygous mating and genotyped by PCR. Their WT littermates were used as controls. These *Nf1*^+/−^ mutant mice do not develop any abnormalities and are commonly used to study the role of neurofibromin. Ras-guanine nucleotide-releasing factor 1 overexpressing (Ras-GRF1 OE) mice, which exhibit a significant increase in p140^Ras−GRF1^ protein (three folds to WT levels), were obtained as previously reported^37,38^. Mice were housed under a 12-h light/dark cycle with *ad libitum* access to food. All experiments were performed in male and female 3–6 month old mice. Experimental protocols were approved by the Cajal Institute Committee on Human and Animal Experimentation and CSIC Ethics Committee, and were in accordance with guidelines of the European Union Council Directive (86/609/European Economic Community).

### 6-OHDA lesion and drug treatment

*Nf1*^+/−^ mice and their WT littermates were anesthetized with 2% v/v isofluorane and received unilateral stereotaxic injections (2 × 2 μl) of 6-OHDA-HBr (5 μg/μl, 20 mM, containing 0.02% ascorbic acid, Sigma-Aldrich, St. Louis, Missouri, USA) in the left striatum at the following coordinates (mm from bregma): AP = +0.65, L = −2 and V = −4 and −3.5, as previously described^8,50^. Ras-GRF1 OE mice and their WT littermates were lesioned by the administration of 1 μl of 6-OHDA-HBr in the right ascending medial forebrain bundle at the following coordinates: AP = −0.7, L = −1.2 and V = −4.7, as described^5^.

#### Establishment of L-DOPA-induced dyskinesia: escalating L-DOPA dosing regimen

After 3 weeks of recovery, a set of *Nf1*^+/−^ and WT animals were treated daily for 9 consecutive days with benserazide hydrochloride (10 mg/kg i.p., Sigma-Aldrich), followed 20 minutes later by administration of L-DOPA methyl ester (Sigma-Aldrich), using an escalating dosing regimen with each dose administered for 3 consecutive days (5, 10 and 20 mg/kg i.p.). Similarly, Ras-GRF1 OE mice and their WT littermates were treated for 9 consecutive days with an escalating L-DOPA dosing regimen (3, 6 and 12 mg/kg i.p.) plus benserazide. In all cases control animals were treated daily with saline.

A second group of hemiparkinsonian *Nf1*^+/−^ mice was used to assess the effect of lovastatin on development of LID. Lovastatin (Mevinolin; Sigma-Aldrich), in the lactone form, was dissolved in ethanol and incubated at 50 °C in 0.1 N NaOH for 2 hours for the conversion to the sodium salt. Volume was adjusted with water and pH was adjusted to 7.5 with HCl^23,51^. 3-weeks after lesion, mice were pretreated with lovastatin (20 mg/kg i.p.) or 0.9% saline for 3 days. Afterwards, for 10 consecutive days, mice received 20 mg/kg of L-DOPA 2.5 h after lovastatin or saline administration.

### Behavioral analysis

#### Locomotor activity and motor coordination

Horizontal and vertical activities, as well as total distance traveled, were recorded in mice as described^52^. Motor coordination was measured using the rotarod (Ugo Basile, Italy) following an accelerating protocol, with increasing speed from 4 to 40 rpm over a 5 minute period as described^53^. Briefly, mice were tested in 6 consecutive trials 20 minutes apart. Motor coordination was tested before 6-OHDA lesion (naïve), 3 weeks after lesion (Parkinsonian state), and during L-DOPA treatment (dyskinetic state, ON L-DOPA) on day 14, 24 hours after the last L-DOPA injection to avoid overtraining and the peak dyskinesia^50,54^.

#### Dyskinetic score

L-DOPA-induced dyskinesia was evaluated using a 0–4 severity scale previously established^5,8,55,56^. Mice were placed in separate glass cylinders and AIMs were assessed daily for 1 minute (monitoring period) every 20 minutes after L-DOPA over a period of 2 h, or on day 13 in the lovastatin experiment. The total AIMs score is the sum of individual scores for each AIM subtype.

### Immunohistochemistry and image analysis

Animals were sacrificed 1 hour after the last L-DOPA injection and perfused and fixed with 4% paraformaldehyde. Brains were cut into 30-µm-thick coronal sections and immunohistochemistry studies were carried out according to published procedures^57–59^ with the following antibodies: anti-tyrosine-hydroxylase (TH) (1:1000, Millipore, Temecula, CA, USA, ref. AB152), FosB (1:7500, Santa Cruz Biotechnology, Santa Cruz, CA, USA, ref. sc-48), pERK1/2 (1:250; Cell Signaling Technology, ref. 9101 L) and phospho-(Ser10)-acetyl-(Lys14)-histone-3 (pAcH3, 1:1500, Upstate, Cell Signaling Solutions, Lake Placid, NY, USA, ref. 07–081).

Quantification of FosB, pAcH3 and pERK1/2 immunoreactivity was carried out by nucleus count with the “cell counter” plug-in in NIH ImageJ software^3,5,11^. The number of immunolabelled nuclei was determined in 5–9 animals per group, using 5 serial rostrocaudal sections per animal and 2 counting frames on the dorsolateral striatum per section (0.091 mm^2^ each frame). Images were digitized with a light microscope (Leica, Heidelberg, Germany) under 40x objective. Before counting, images were threshold at a standardized gray-scale level, empirically determined by 2 different observers to allow detection of stained nuclei from low to high intensity, with suppression of very lightly stained nuclei. The data are presented as number of stained nuclei per mm^2^ (mean ± SEM).

Quantification of TH immunoreactivity fibers in the striatum was performed with the NIH ImageJ software as previously described^13,60^, analyzing 6–9 rostrocaudal striatal sections per animal. Briefly, 4x lens color images were converted into a gray scale and the area of staining (i.e., denervated area) was quantified as percentage of the total striatal area. Data are presented as the percentage of depletion of dopaminergic fibers.

### cAMP and Ras-GTP assay

All assays were performed on dissected striatum, snap-frozen in liquid nitrogen. cAMP levels were quantitated from tissue homogenized in 0.1 M HCl, using a cAMP HTRF kit (CisBio) following manufacturer’s instructions.

Active Ras (Ras-GTP) was detected by Raf1-RBD immunoprecipitation using the RAS activation kit (Millipore). Briefly 500 µl of homogenized extract were incubated with Raf-1 RBD and agarose for 45 min at 4 °C. Then samples were centrifuged for 10 seconds at 14000 × g and agarose pellets were washed three times in the same conditions. Pellets were resuspended in 40 µL of 2X Laemli buffer supplemented with 2 mM DTT and boiled for 5 min at 100 °C. Samples were then processed for WB by following manufacturer’s instructions. Total Ras was also detected as loading control. Both cAMP and Ras-GTP levels were normalized to the levels observed in the striatum of the WT group.

### Statistical analysis

Both the behavioral and immunolabeling quantification data of L-DOPA ascending-dose experiment were analyzed by one-way ANOVA. cAMP and Ras-GTP levels data were evaluated by unpaired t-test. Lovastatin experiment data were analyzed by two-way ANOVA followed by Student-Newman-Keuls post hoc test. Differences were considered statistically significant at *p* < 0.05.

## Electronic supplementary material

Supplementary Figure S1

## Data Availability

The data that support the findings of this study are available from the corresponding author upon reasonable request.
