# Supplementary material for: Genetic enhancement of Ras-ERK pathway does not aggravate L-DOPA-induced dyskinesia in mice but prevents the decrease induced by lovastatin
Source: Sci Rep. 2018 Oct 18;8:15381. doi: 10.1038/s41598-018-33713-3 (PMC6194127; doi:10.1038/s41598-018-33713-3)

Supplementary information: Manuscript reference **SREP-18-19417A**

Genetic enhancement of Ras-ERK pathway does not aggravate L-DOPA-induced dyskinesia in mice but prevents the decrease induced by lovastatin. Irene Ruiz De Diego, Stefania Fasano, Oscar Solís, José-Rubén Garcia-Montes, José M. Brea, María I. Loza, Riccardo Brambilla, Rosario Moratalla.

Supplementary Figure S1: Expanded Blots shown in main Fig 1E

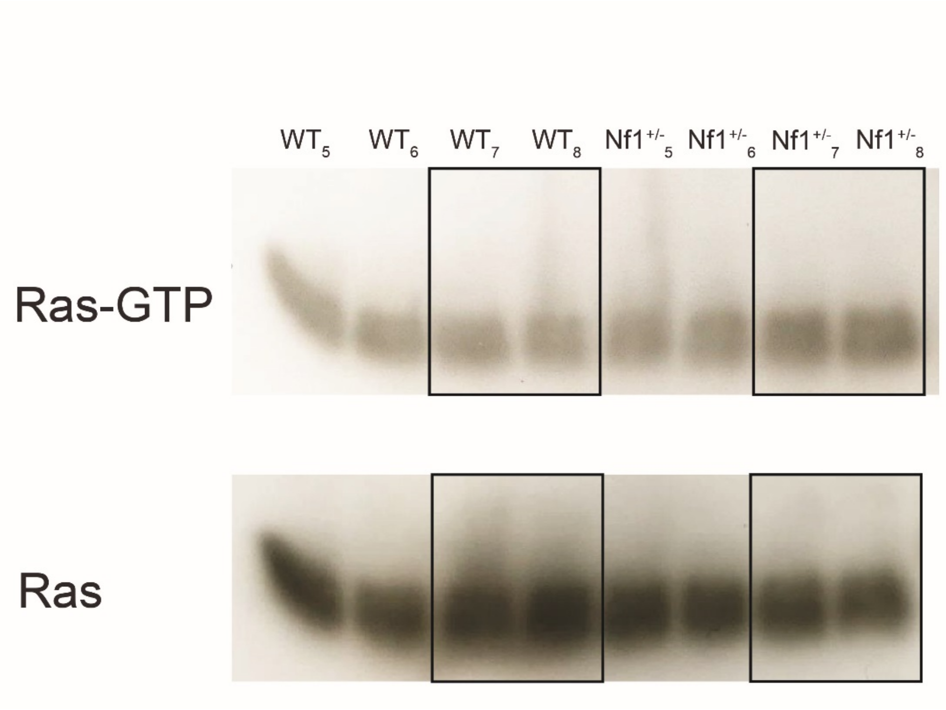

Supplement: Supplementary file 1 — Supplementary Figure S1 [file 41598_2018_33713_MOESM1_ESM.pdf]
